# Advancing Pediatric Growth Assessment with Machine Learning: Overcoming Challenges in Early Diagnosis and Monitoring

**DOI:** 10.3390/children12030317

**Published:** 2025-02-28

**Authors:** Mauro Rodriguez-Marin, Luis Gustavo Orozco-Alatorre

**Affiliations:** 1 Departament of Marketing and Analysis, Tecnologico de Monterrey Campus Guadalajara, Zapopan 45201, Mexico; 2 Hospital Civil de Guadalajara “Dr. Juan I. Menchaca”, Universidad de Guadalajara, Guadalajara 44100, Mexico; gustavo.orozco@cucs.udg.mx

**Keywords:** pediatric growth anomalies, health, logistic regression, machine learning (ML), Artificial Intelligence (AI), children

## Abstract

Background: Pediatric growth assessment is crucial for early diagnosis and intervention in growth disorders. Traditional methods often lack accuracy and real-time decision-making capabilities This study explores the application of machine learning (ML), particularly logistic regression, to improve diagnostic precision and timeliness in pediatric growth assessment. Logistic regression is a reliable and easily interpretable model for detecting growth abnormalities in children. Unlike complex machine learning models, it offers parsimony in transparency, efficiency, and reproducibility, making it ideal for clinical settings where explainable, data-driven decisions are essential. Methods: A logistic regression model was developed using R to analyze biometric and demographic data from a cross-sectional dataset, including real-world data from public institucions. The study employed a bibliometric analysis to identify key trends and incorporated data preprocessing techniques such as cleaning, imputation, and feature selection to enhance model performance. Performance metrics, including accuracy, sensitivity, and the Receiver Operating Characteristic (ROC) curve, were utilized for evaluation. Results: The logistic regression model demonstrated an accuracy of 94.65% and a sensitivity of 91.03%, significantly improving the identification of growth anomalies compared to conventional assessment methods. The model’s ROC curve showed an area under the curve (AUC) of 0.96, indicating excellent predictive capability. Findings highlight ML’s potential in automating pediatric growth monitoring and supporting clinical decision-making, as it can be very simple and highly interpretable in clinical practice. Conclusions: ML, particularly logistic regression, offers a promising tool for pediatric healthcare by enhancing diagnostic precision and operational efficiency. Despite these advancements, challenges remain regarding data quality, clinical integration, and privacy concerns. Future research should focus on expanding dataset diversity, improving model interpretability, and conducting external validation to facilitate broader clinical adoption.

## 1. Introduction

Accurate and timely assessment of pediatric growth is crucial for early diagnosis and effective monitoring of various health conditions. Traditional methods of pediatric growth assessment, such as the Tanner–Whitehouse and Bayley–Pinneau methods, rely on current height, skeletal maturation indices, and growth charts. These methods have inherent limitations in their ability to accurately predict adult height and identify growth disorders in a timely manner [1]. The standard errors associated with these traditional methods range from 5 to 6 cm, which can lead to inaccuracies in growth assessment, particularly during the onset of puberty [2]. These constraints hinder real-time decision-making and necessitate the exploration of more precise predictive approaches.

The rapid advancements in machine learning (ML) offer promising solutions to overcome these challenges, positioning ML as a transformative tool in pediatric growth assessment. ML aids in the early diagnosis and monitoring of growth disorders by leveraging comprehensive datasets and advanced algorithms. Predictive models can identify intricate relationships among biological parameters, facilitating precise and personalized growth assessments [3]. Additionally, the integration of electronic health records with high-volume data streams from primary registries enhances the accuracy and efficiency of ML-based tools, making them effective in both clinical and research applications.

AI-based tools have demonstrated their ability to improve pediatric disease diagnosis, prognosis, and management, addressing critical gaps in pediatric evidence and the shortage of subspecialists [4]. Notable applications include bone age assessment (BAA), where neural networks improve accuracy and reduce complexity [5,6] and the use of deep learning models like VGG16, which outperform traditional methods in BAA. Furthermore, Random Forest models have shown effectiveness in predicting adult height [3,7].

The proposed logistic regression algorithm exemplifies how ML advancements can bridge critical gaps in pediatric healthcare. Utilizing clinical and biometric data, this algorithm identifies patterns indicative of growth abnormalities. Developed with the R programming language, it integrates diverse data sources such as electronic medical records and anthropometric measurements to deliver precise recommendations for evaluation and treatment. This open-access, reproducible research focuses on children aged 6 to 13 years but is adaptable to other age ranges and cohorts, making it versatile for broader applications. However, challenges remain, including the need for large, well-annotated datasets, data privacy and security considerations, and the need for clinical interpretability [8,9].

Logistic regression is a powerful and interpretable statistical modeling technique that provides a structured and reliable framework for identifying growth abnormalities in children. Unlike more complex machine learning (ML) models such as deep learning, logistic regression excels in scenarios where transparency, interpretability, and efficiency are crucial especially in clinical settings where decisions must be explainable and reproducible [2].

Additionally, logistic regression requires significantly less computational power and energy compared to more sophisticated ML techniques, making it ideal for deployment in healthcare environments where real-time processing and resource efficiency are essential [10]. Furthermore, its probabilistic output enables clinicians to assign confidence levels to each prediction, improving medical decision-making and risk assessment.

While advanced models such as neural networks and ensemble learning methods (e.g., Random Forest) have shown promise in growth prediction, their reliance on vast amounts of annotated data, potential overfitting, and lack of interpretability present challenges for clinical implementation [11]. Logistic regression, in contrast, balances accuracy, simplicity, and clinical applicability, making it a highly effective tool for early diagnosis and intervention in pediatric growth monitoring [1].

By leveraging logistic regression in this study, we provide a scalable, interpretable, and statistically sound approach to improving pediatric health outcomes. Future work may explore hybrid models that combine logistic regression with other ML techniques to further enhance predictive performance while preserving interpretability in clinical practice [12].

Moreover, ML can leverage data on nutrition, physical activity, medical history, and socio-demographics to develop risk prediction models, aiding clinicians in decision-making [13]. These tools enhance real-time decision-making, streamline screening processes, and support personalized preventive strategies in various healthcare domains, including precision dentistry [14].

The integration of ML into pediatric growth assessment enhances diagnostic accuracy, automates data analysis, and facilitates personalized treatment protocols [15]. Despite the existing challenges, ML holds immense potential to revolutionize pediatric healthcare, improving diagnostic precision, operational efficiency, and the overall health and well-being of children [3].

Research Questions:

Q1: To what extent does the implementation of logistic regression algorithms enhance the interpretability of early diagnosis in the clinical practice of pediatric growth disorders compared to other ML methods?

Hypothesis:

H1: The implementation of logistic regression algorithms significantly enhances the interpretability of early diagnosis in the clinical practice of pediatric growth disorders compared to other ML methods, due to their transparency, ease of interpretation, parsimony and suitability for clinical decision-making.

## 2. Bibliometrics Analysis

This study began with a bibliometrics analysis, leveraging the VOSviewer 1.6.19 tool [16,17] and the SCOPUS database to map the evolving landscape of research on machine learning (ML), artificial intelligence (AI), Logistic, Logistic Regression (LR), and related technologies in pediatric growth. The findings reveal a striking global surge in publications addressing these topics, underscoring the growing interest and investment in AI-driven solutions for pediatric healthcare.

**Figure 1:**
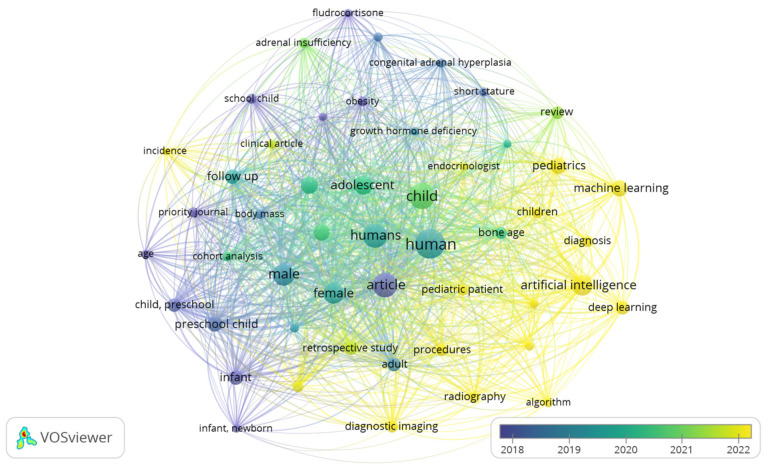
Network Visualization (Pediatric growth, ML, LR, AI_SCOPUS).

**Figure 2:**
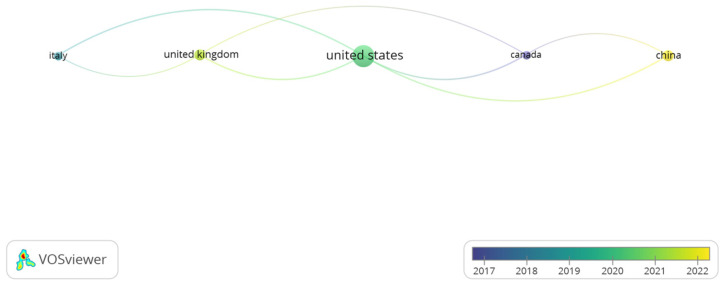
Countries that have conducted the most research (Pediatric growth, ML, LR, AI_SCOPUS).

The descriptive analyses of the collected literature highlight key trends and patterns, visualized in Figure 1 and Figure 2. Figure 1 depicts a bibliometric network visualization generated with VOSviewer, highlighting research trends in pediatric growth, machine learning (ML), linear regression, and artificial intelligence (AI) over recent years. These visuals illustrate not only the exponential rise in research output but also the expanding geographical (Figure 2) and interdisciplinary reach of studies in this field. The data suggest that ML and AI are increasingly recognized as transformative tools for understanding and addressing pediatric growth challenges, from early diagnosis to personalized treatment strategies.

This section delves into the quantitative and qualitative dimensions of the literature, offering a comprehensive overview of how AI and ML are reshaping pediatric growth research and practice worldwide.

Between 2018 and 2024, the scientific literature on pediatric growth showed considerable progress, highlighting the application of emerging technologies such as artificial intelligence (AI) and machine learning (ML). These tools have been integrated into diagnosis, growth prediction, and bone age assessment, optimizing precision and personalization in pediatric care. Among ML models, logistic regression has demonstrated a fundamental role in predicting clinical outcomes due to its ability to model relationships between growth variables and health outcomes. However, there remains a need for more publications that delve into its application, external validation, and clinical utility in various pediatric contexts.

In addition, traditional approaches focused on epidemiological factors such as obesity, body mass index, and longitudinal follow-up persist, along with the study of endocrine disorders that affect growth, such as congenital adrenal hyperplasia. The convergence of these traditional approaches with the use of ML, particularly logistic regression, represents a key opportunity to improve risk prediction, early diagnosis, and clinical decision-making in pediatrics, highlighting the importance of continued research in this area.

Between 2017 and 2022, the United States led research on delayed growth treatment in children, followed by the United Kingdom, Italy, Canada, and China. The evolution of this research reflects a shift from traditional clinical approaches to more advanced methods, integrating biotechnology and computational tools. Medicine remains the dominant discipline, with significant contributions from biochemistry, genetics, and computer sciences. This multidisciplinary approach has enhanced the understanding of growth disorders, promoting personalized treatments through genetic analysis and artificial intelligence, which have become increasingly relevant in recent years.

**Figure 3:**
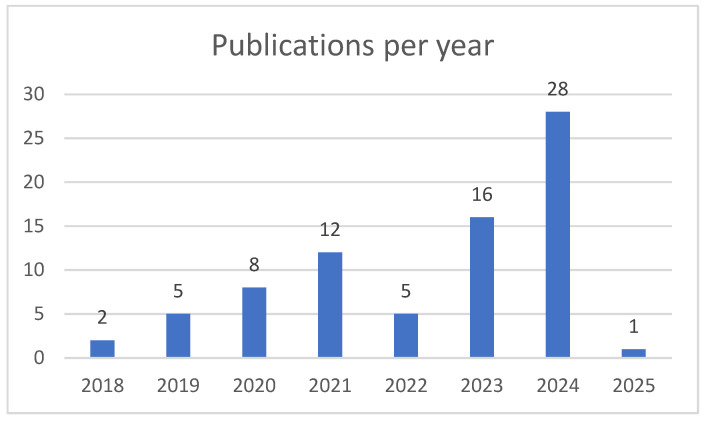
Number of publications per year on the topic (Pediatric growth, ML, LR, AI_SCOPUS).

On the other hand, the evolution of publications related to the analysis and prediction of pediatric growth using ML and LR demonstrates a significant upward trend over the past years. As shown in Figure 3, there has been a steady increase in the number of publications, starting from just two articles in 2018 and rising to five in 2019. This growth continued with 8 publications in 2020 and a notable peak of 12 in 2021, reflecting an increasing interest in applying advanced statistical models in pediatric research.

Although there was a slight decline in 2022 with 5 publications, interest surged again in 2023, reaching 16 publications, and dramatically peaking in 2024 with 28 publications, the highest number recorded during the period analyzed. This growth underscores the expanding role of LR in pediatric growth studies, particularly for risk prediction, growth trend analysis, and early detection of developmental disorders. The slight drop to one publication in 2025 may reflect incomplete data for the year.

In conclusion, the data highlight a clear trend of growing interest and application of ML and LR in pediatric growth prediction, driven by the increasing need for accurate, data-driven clinical decision-making tools in pediatric healthcare.

With this preamble, it is evident that this topic remains underexplored, representing an opportunity to delve deeper into it in the coming years, particularly considering new technological tools such as generative AI. These tools will support efforts to improve the health of children. While the United States, the United Kingdom, Italy, Canada, and China have led research on delayed growth treatment, there is still room to expand knowledge, especially through interdisciplinary collaboration. The integration of medicine, biochemistry, genetics, and computer sciences has already advanced understanding, but emerging technologies, such as AI, offer new possibilities for personalized diagnosis and treatment. In this sense, the present research serves as an early alert to help close existing gaps in the various contexts where machine learning (ML) can be applied. This shift from traditional approaches to data-driven models could revolutionize pediatric growth management, ensuring more precise, early interventions. In conclusion, leveraging technological innovations and fostering global research collaborations will be key to addressing current gaps, ultimately improving health outcomes for children with growth disorders.

## 3. Literature Review

The field of pediatric growth has undergone a transformative shift with the integration of machine learning (ML) and artificial intelligence (AI) technologies. This literature review synthesizes recent advancements in ML applications for pediatric growth assessment, highlighting their potential to revolutionize clinical practice, enhance diagnostic accuracy, and improve patient outcomes.

Recent studies have demonstrated the efficacy of ML in pediatric growth assessment, particularly in predicting adult height and identifying growth-related risks. Refs. [3,13,18] explored the application of ML algorithms to analyze growth patterns, leveraging large datasets to predict outcomes with remarkable precision. These tools provide clinicians with actionable insights and enable early intervention in cases of growth disorders, ensuring timely and effective treatment.

Beyond growth assessment, AI’s role in pediatric disease diagnosis has shown transformative potential. A large-scale study by [19] developed a deep learning system using millions of electronic medical records from a childcare center in Shanghai, achieving a diagnostic concordance rate of over 93%, surpassing human diagnosis in many cases. This underscores AI’s capacity to enhance diagnostic accuracy, reduce errors, and streamline clinical workflows in pediatric care.

AI applications in pediatrics extend across various specialties, including precision medicine, cardiology, radiology, and neurology. Ref. [20] highlights AI’s growing accuracy and efficiency in diagnostics and patient monitoring. AI-powered tools analyze imaging data, predict disease progression, and personalize treatment plans, marking a significant advancement toward precision medicine in pediatric healthcare.

Despite its transformative potential, integrating AI into pediatric medicine presents challenges. Ref. [21] identifies barriers such as data security concerns, validation challenges, and the need for explainability in AI-driven decisions. Addressing these issues is vital to build clinician trust and ensure ethical AI use in pediatric healthcare. Robust validation frameworks and interdisciplinary collaboration are crucial to fully realize AI’s potential in this field.

Looking ahead, the convergence of ML and pediatric growth assessment represents a paradigm shift in healthcare. As AI technologies evolve, their applications in pediatrics are expected to expand, offering new opportunities for early diagnosis, personalized treatment, and improved patient outcomes. Ref. [22] highlights the growing use of supervised ML in pediatric critical care, emphasizing predictive models to improve clinical outcomes. Implementing AI in pediatric healthcare requires addressing challenges such as dynamic growth patterns, data scarcity, and model explainability. Ethical considerations include preventing bias, ensuring informed consent, and protecting vulnerable populations. Collaboration among clinicians, data scientists, and ethicists is essential to develop transparent, equitable, and trustworthy AI solutions that prioritize children’s well-being [23].

The application of ML and AI in pediatric growth assessment and healthcare is rapidly advancing with immense potential. From predicting adult height to diagnosing complex diseases, these technologies are reshaping pediatric medicine. While challenges remain, the opportunities for innovation and improvement are vast, paving the way for a future where AI-powered tools are integral to pediatric care. As researchers and clinicians continue exploring these possibilities, the focus must remain on ensuring the ethical, secure, and effective use of AI to benefit pediatric patients worldwide.

## 4. Methods

This study employed a bibliometric analysis to explore the application of machine learning (ML) and artificial intelligence (AI) in pediatric growth assessment. A comprehensive search strategy was designed using the SCOPUS database with keywords such as “pediatric growth”, “ML”, and “AI”, refined through Boolean operators. To visualize keyword co-occurrence, research trends, and emerging themes, the VOSviewer tool was employed for bibliometric analysis, highlighting global contributions and interdisciplinary developments.

To enhance the depth of the analysis, a subset of studies underwent in-depth case reviews to assess methodological rigor and relevance. Complementing the insights from VOSviewer, a custom algorithm developed in R automated tasks such as data cleaning, keyword extraction, and trend analysis. This integration of bibliometric analysis with R-based algorithmic processing provided a robust, multi-dimensional understanding of the field, revealing a significant increase in global research output on ML and AI applications in pediatric growth. The methodology ensured both the reliability and impact of the findings, offering actionable insights for future research and clinical applications.

In the context of this study, a machine learning algorithm, specifically logistic regression, was developed using R software.4.2.3 version The algorithm is designed to predict growth-related risks in pediatric populations, focusing on identifying children at risk of stunted growth or abnormal height variations relative to their age. Leveraging comprehensive datasets, including publicly available data from Stanford Medicine Children’s Health (https://www.Stanfordchildrens.org/es, accessed on 7 July 2024), the model ensures data privacy and ethical integrity while providing a robust foundation for accurate predictions.

The development process integrated key anthropometric variables such as age, height, and growth patterns, along with demographic and socio-environmental factors. Multiple pilot tests were conducted to calibrate the algorithm, ensuring its reliability and performance across diverse data subsets. Throughout the development of the model, a total of twelve tests were conducted to adjust the parameters and fine-tune the algorithm. Additionally, the use of cross-validation techniques, specifically k-fold cross-validation, has been implemented and described to assess the robustness of the model and mitigate overfitting [24]. These tests refined the model’s predictive accuracy, enabling it to detect growth deviations effectively and support early diagnosis and intervention strategies. The logistic regression model evaluates growth trends, compares them with standardized growth charts, and identifies outliers that may indicate underlying health issues. In the study, the mean height per classification was used as an imputation method to handle missing values and mitigate inconsistencies in the data. This approach, although simple, helps maintain the generality of the model by assigning values based on the central tendency of each group. However, it is important to note that this method assumes that missing data are completely random (MCAR), which may not always be valid. Aligned with open science principles, the logistic regression algorithm is publicly accessible through a dedicated GitHub repository. This open-access approach promotes transparency, reproducibility, and collaborative development within the scientific community. Researchers and healthcare professionals can utilize, adapt, and improve the algorithm for various applications, including pediatric growth monitoring programs, clinical settings, and public health initiatives. By making the algorithm freely available, the study aims to contribute to global efforts in enhancing early detection and management of growth-related conditions in children [25].

### 4.1. Machine Learning

Machine learning (ML) relies on the use of data that include both the desired outcomes and relevant features for predicting those outcomes. The primary objective is to design algorithms capable of processing these features to generate accurate predictions when the outcome is unknown. This process involves training algorithms on historical datasets with known outcomes, enabling the identification of significant patterns and relationships for future application in new scenarios.

ML algorithms have demonstrated effectiveness across diverse fields. In healthcare, they enhance disease prediction and diagnostic accuracy [26,27]. In finance, ML supports risk assessment by identifying trends and potential vulnerabilities [28], while in sports, it forecasts outcomes with high precision [27]. These models not only improve decision-making in real time but also continuously adapt and learn from new data, optimizing their performance over time.

The success of ML models lies in their ability to generalize from previously seen data, making them valuable tools for solving complex problems. Recent studies emphasize the critical role of feature selection and algorithm optimization in enhancing predictive accuracy across various contexts [29]. This adaptability has transformed problem-solving approaches across industries, positioning ML as an indispensable tool in the digital era.

### 4.2. Logistic Regression

Logistic regression is a statistical method used to model the probability of a binary outcome, where the result can take only two possible states, such as “yes” or “no”, “success” or “failure”, or “0” and “1”. It is particularly effective in classification problems within statistics and machine learning due to its ability to model relationships between a dependent binary variable and one or more independent variables through the logistic function. This function, also known as the sigmoid function, transforms any input value into a probability between 0 and 1, making it ideal for representing probabilities [1].

Logistic regression stands out as a machine learning model with multiple advantages, particularly in terms of interpretability, parsimony, and speed. Unlike more complex models such as neural networks or random forests, logistic regression offers high interpretability thanks to its coefficients, which allow for an easy understanding of the relationship between predictor variables and the target variable. This is particularly useful in applications where model transparency is crucial, such as in medicine or finance. Additionally, its parsimony, that is, its ability to provide simple and efficient solutions makes it an ideal choice for small or medium-sized datasets, where more complex models might overfit or require higher computational costs. In terms of speed, logistic regression is notably faster to train compared to models like support vector machines (SVM) or neural networks, making it suitable for applications that require real-time results or have limited resources. In summary, logistic regression combines simplicity, efficiency, and clarity, making it an invaluable tool in real-world clinical practice. Table 1, this comparison highlights why logistic regression is particularly suitable for scenarios where interpretability and ease of implementation are crucial, such as clinical decision-making in pediatric growth assessment.

Logistic regression has demonstrated versatility across various domains. In healthcare, it is widely applied for disease prediction, showing performance comparable to more complex machine learning models in predicting chronic conditions such as diabetes and cardiovascular diseases [30]. Despite the rise in advanced machine learning models like neural networks and gradient boosting machines, logistic regression remains highly relevant due to its simplicity, interpretability, and robustness. Its capability to handle multicollinearity, ease of implementation, and efficiency with small datasets make it particularly valuable in clinical and industrial settings where decision transparency is critical [31].

The model’s coefficients, or weights, are typically estimated using the maximum likelihood method, which seeks to maximize the probability of observing the given data. Each coefficient represents the change in the log odds of the outcome for a one-unit increase in the corresponding predictor variable, holding all other variables constant. A positive coefficient indicates an increase in the log odds of the event occurring, while a negative coefficient suggests a decreased likelihood.

Recent studies highlight the utility of marginal effects, which offer direct interpretations on the probability scale, making them especially useful for policy-related and clinical decision-making models [32]. Additionally, graphical methods, such as plotting predicted probabilities, enhance the understanding of interaction effects, providing clearer insights into how predictors influence outcomes under varying conditions [33].

Logistic regression continues to play a crucial role in healthcare and medical research, where it is extensively used for predictive modeling. Its balance of interpretability, efficiency, and robustness ensures its ongoing relevance, even as more complex machine learning techniques emerge.

### 4.3. Problem Identification

The first step is to identify a healthcare challenge or problem where machine learning, in particular logistic regression, can be applied [34]. This could range from diagnosing diseases and personalizing treatment to predicting patient outcomes. The key is to define a clear and specific problem where machine learning can add value. In the case of this article, the intention is to detect in a timely manner the presence of any anomaly in the average linear growth of children according to their age. If it is confirmed and evaluated by the doctor, recommendations will be made [35]. Although longitudinal studies of growth and development constitute the ideal method to describe the magnitude and speed of growth [36], failing that, different mathematical models can be used to understand the variations in growth in height of a population through specific functions in cross-sectional studies [37]. The Preece–Baines 1 (PB1) model is adapted to the study of height growth and has been applied to describe it in cross-sectional samples from childhood to the end of adolescence. PB1 includes mathematical and biological parameters to determine the age of occurrence, magnitude, and speed of growth during the different stages of development until reaching adult size [38]. In the present study, an ML model is built and adapted to the database, specifically the Logistic Regression model, the programming of the algorithm is executed in the R programming system. The data source will be a cross-sectional study collecting demographic information, evolution, and comparison of the height of infants of both sexes from 6 to 13 years old. This study will use data published by Stanford Medicine Children’s health. See Table 2 and Table 3.

The data presented in Table 2 outline the height ranges by age and gender for children aged 6 to 13 years, sourced from publicly available data in the Stanford Medicine Children’s Health Repository. This table provides a benchmark for assessing typical growth trajectories, offering minimum and maximum height values for both girls and boys across different age groups. Notably, all genders exhibit a progressive increase in height with age, consistent with established pediatric growth patterns. For instance, girls’ heights range from 106.68 cm at age 6 to 167.16 cm at age 13, while boys show a similar trajectory from 106.68 cm to 169.37 cm within the same age span.

These reference values are critical in the context of the present study, as they allow for comparative analysis with the pilot dataset of 1000 children. The pilot data served as baseline ranges to detect children with short stature and classify them according to the reference database used. By juxtaposing the collected data with these standardized growth ranges, it becomes possible to identify deviations from typical growth patterns, such as stunted growth or early-onset growth spurts. Such deviations could signal underlying health conditions, nutritional deficiencies, or genetic factors that warrant further investigation.

Moreover, the slight differences observed between boys and girls, particularly in the later years (ages 11–13), highlight the impact of puberty-related growth spurts, which tend to occur earlier in girls than in boys. The ability to detect and analyze these variations underscores the relevance of using robust, standardized datasets like those from Stanford Medicine Children’s Health. This approach not only enhances the accuracy of growth assessments but also supports the development of predictive models to identify growth anomalies with greater precision in diverse pediatric populations.

The findings from the test research data, as presented in Table 3, confirm typical growth patterns in pediatric populations, as evidenced by the data analyzed in this study. The variance in height (209.25) and the standard deviation (14.46 cm) indicates moderate dispersion, consistent with the expected diversity in a heterogeneous group. Additionally, the skewness and kurtosis suggest a slightly skewed distribution, which could be attributed to genetic, nutritional, or socioeconomic factors.

The relationship between age and height aligns closely with standardized growth curves; however, the presence of outliers may warrant individual case studies to explore specific growth anomalies [39]. This aspect is where the present research proves particularly pivotal, as it focuses on identifying children exhibiting stunted growth relative to their age.

A significant strength of this study lies in its reproducibility. The methodology and algorithms developed are designed to be generalizable and can be applied to diverse datasets, including those from individual pediatricians, hospitals, states, countries, or regions, depending on the scope of the investigation [40]. This adaptability enhances the potential impact of the research, making it a valuable tool for broader applications in pediatric growth monitoring.

However, it is crucial to acknowledge that some datasets may contain sensitive information. Ethical considerations and secure data handling practices are essential to ensure the responsible use of such data. This includes implementing robust privacy measures and compliance with data protection regulations to maintain the integrity and confidentiality of patient information [41].

The algorithm developed in this study offers a precise and efficient tool for identifying and addressing growth anomalies, contributing significantly to the field of pediatric health and development.

**Figure 4:**
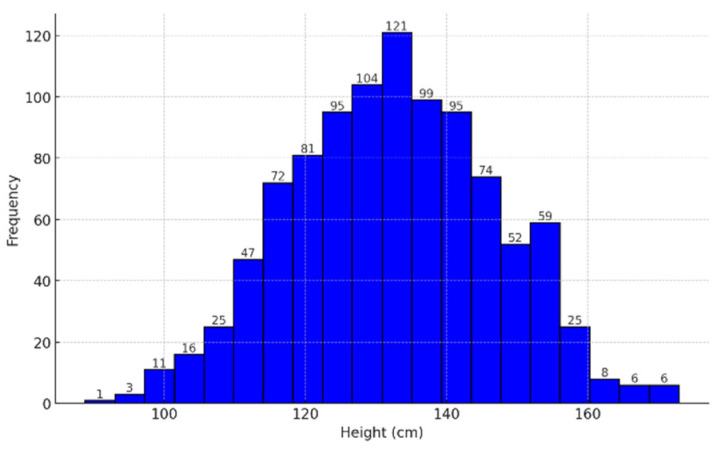
Histogram of height in centimeters.

**Figure 5:**
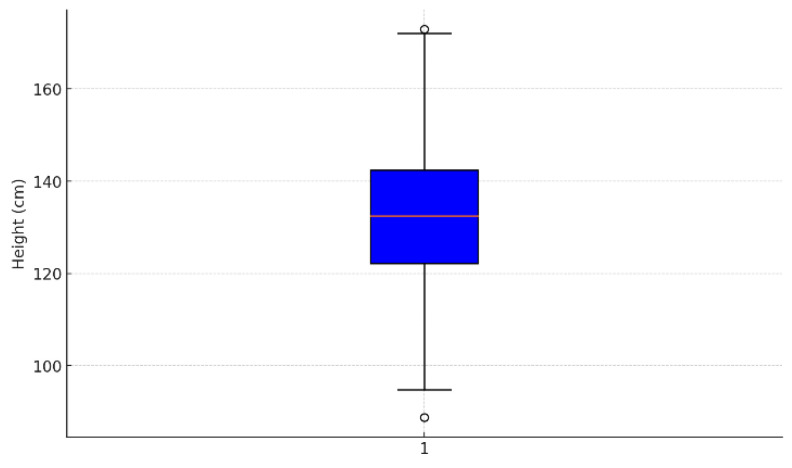
Figure Boxplot age and Heigth.

**Figure 6:**
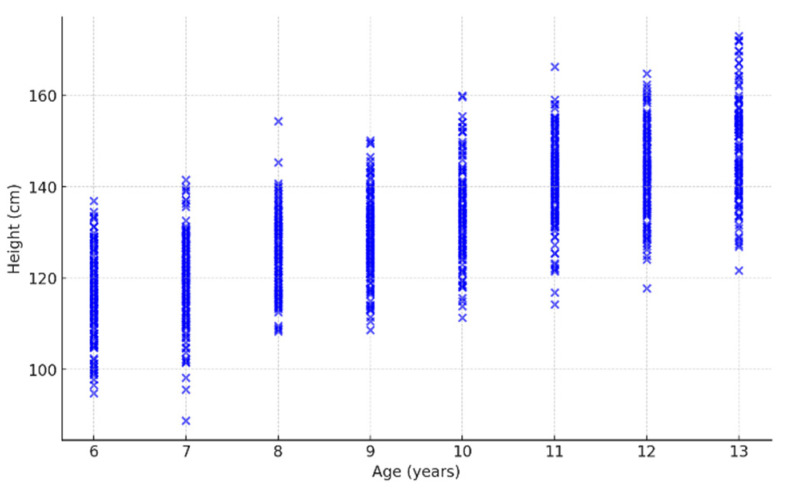
Relationship Between Age and Height.

Figure 4, Figure 5 and Figure 6 presented are integral to the methodology employed in this study, providing the statistical foundation necessary for applying logistic regression in the diagnosis and monitoring of children’s stature. The histogram illustrates the distribution of height within the sample, revealing a normal distribution pattern centered around 132.5 cm, with frequencies peaking in this range. This distribution is essential for understanding the overall growth trends and identifying any deviations that may signify growth disorders. The boxplot complements this analysis by highlighting the central tendency, variability, and the presence of outliers’ key factors that influence the predictive power of logistic regression models. The identification of outliers allows for the isolation of cases that may require more detailed clinical evaluation.

The scatter plot showing the relationship between age and height further enhances the robustness of the logistic regression model. This visual representation of data points reveals a clear positive correlation between age and height, a critical predictor when developing growth assessment algorithms. By incorporating these statistical analyses into the logistic regression framework, the study can more accurately classify children at risk of growth abnormalities [42]. The integration of these figures into the methodology not only strengthens the diagnostic precision but also facilitates continuous monitoring, enabling early intervention strategies in pediatric healthcare. This comprehensive approach underscores the potential of data-driven models in enhancing the effectiveness of growth assessments and personalized medical care for children.

Raw data often contain inconsistencies, missing values, and errors, which can compromise the reliability of analytical models [43]. As part of this study, data preprocessing was conducted to clean and transform the raw data into a format suitable for machine learning (ML) algorithms. This process included handling missing values, correcting data inconsistencies, and normalizing variables. Preprocessing is a critical step, as the accuracy and performance of ML models are directly influenced by the quality of the data input.

Variable selection is equally essential in optimizing ML models. In this study, key variables were identified from the existing dataset, focusing on those most relevant to pediatric growth assessment, such as age, height, and growth patterns. This step is particularly crucial in healthcare, where selecting the right features can significantly impact diagnostic accuracy and treatment outcomes. Furthermore, the methodology is adaptable to different populations, allowing for its application in various settings, such as schools, regions, or even nationwide health programs. It is important to note that results may vary depending on the demographic and environmental characteristics of the population studied.

To ensure data security and protect sensitive information, the study utilized publicly available data from reputable sources, such as Stanford Medicine Children’s Health. This approach minimizes the risk associated with handling sensitive health data from pediatric institutions while maintaining the integrity and reliability of the analysis. Using public datasets not only strengthens the scientific rigor of the study but also facilitates its broader dissemination without ethical or privacy concerns, contributing to the advancement of pediatric growth monitoring and early diagnosis practices.

### 4.4. Data Collection and Preprocessing

The study utilizes a cross-sectional dataset comprising real-world data published by Stanford Medicine Children’s Health. This publicly available information serves as the foundation for the analysis, ensuring data integrity while safeguarding sensitive health information. By relying on real data from reputable sources, the study maintains scientific rigor without compromising the privacy of pediatric populations.

#### 4.4.1. Dataset Description

The dataset consists of 1000 records and 3 variables:

Age: An integer numeric variable representing the age of the children, with values ranging from 6 to 13 years.

Height: A continuous numeric variable indicating height in centimeters.

Height_Condition: A binary categorical variable indicating whether the height falls within the normal range (1 = appropriate height for age; 0 = short stature).

#### 4.4.2. Variable Classification

Numéric

- Age
- Height

Categoric

- Height_Condition (binary)

#### 4.4.3. Variable Analysis

Target Variable:

Height_Condition is the target variable, as the model is designed to predict whether a child has an appropriate height or not.

Features (Independent Variables):

- Age
- Height

Metavariables:

There are no explicit metavariables in the dataset, but others, such as gender or socioeconomic factors, could be integrated if the dataset were expanded.

Raw data undergo a thorough preprocessing phase, which includes data cleaning, addressing missing values, and transforming the information into a format suitable for machine learning (ML) models. This step is crucial, as the quality of the input data directly impacts the accuracy and performance of the models. The variables selected for the ML model encompass demographic, nutritional, physical activity, and socio-demographic data, all of which are critical for understanding growth patterns and identifying potential growth anomalies. This approach strengthens the reliability of the findings and supports the application of ML in pediatric growth monitoring and early diagnosis.

### 4.5. Model Evaluation

Logistic regression is a widely used statistical method for binary classification problems. To assess its performance, several evaluation metrics are employed, each providing unique insights into the model’s effectiveness. Below, we discuss key metrics, including the confusion matrix, accuracy, specificity, and others, which are essential for evaluating logistic regression models.

#### 4.5.1. Confusion Matrix

**Figure 7:**
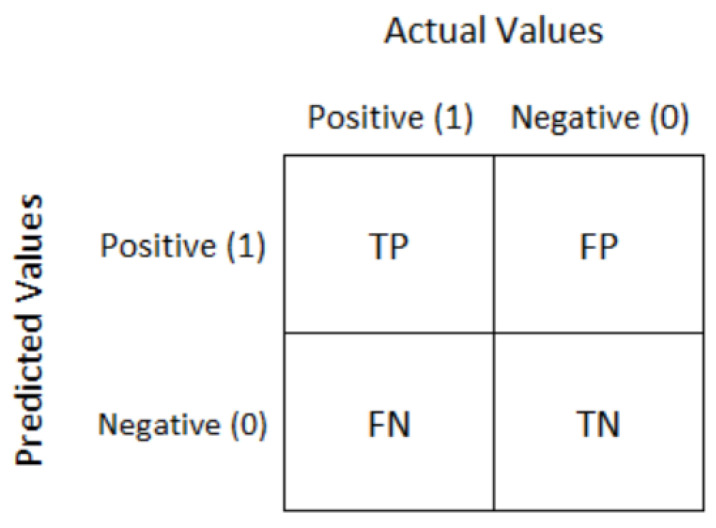
Confusion Matrix Actual Values.

The confusion matrix is a fundamental tool for evaluating classification models. It provides a detailed breakdown of the model’s predictions compared to the actual outcomes. For a binary classification problem, the matrix is structured as follows (see Table 4 and Figure 7).

- True Positives (TP): Cases correctly predicted as positive.
- False Positives (FP): Cases incorrectly predicted as positive (Type I error).
- True Negatives (TN): Cases correctly predicted as negative.
- False Negatives (FN): Cases incorrectly predicted as negative (Type II error).

The confusion matrix serves as the foundation for calculating other performance metrics.

#### 4.5.2. Accuracy

Accuracy measures the proportion of correctly classified instances out of the total number of instances. It is calculated as:

$$Accuracy=\frac{TP+TN}{TP+TN+FP+FN}$$

- Interpretation: High accuracy indicates that the model is performing well overall. However, accuracy alone can be misleading in imbalanced datasets, where one class significantly outweighs the other.

#### 4.5.3. Precision

Precision evaluates the proportion of true positive predictions among all positive predictions. It is calculated as follows.

$$Precision=\frac{TP}{TP+FP}$$

- Interpretation: High precision indicates that the model is effective at minimizing false positives, which is crucial in scenarios where false alarms are costly (e.g., medical diagnoses).

#### 4.5.4. Recall (Sensitivity)

Recall, also known as sensitivity, measures the proportion of true positives correctly identified by the model. It is calculated as follows.

$$Recall=\frac{TP}{TP+FN}$$

- Interpretation: High recall indicates that the model is effective at capturing most of the positive cases, which is important in scenarios where missing a positive case is costly (e.g., fraud detection).

#### 4.5.5. Specificity

Specificity measures the proportion of true negatives correctly identified by the model. It is calculated as follows.

$$\mathrm{Specificity}=\frac{TN}{TN+FP}$$

- Interpretation: High specificity indicates that the model is effective at minimizing false positives for the negative class, which is important in scenarios where correctly identifying negatives is critical (e.g., disease screening).

#### 4.5.6. F1-Score

The F1-score is the harmonic mean of precision and recall, providing a balanced measure of the model’s performance. It is calculated as:

$$F1-score=\frac{2*Precision*Recall}{Precision+Recall}$$

- Interpretation: The F1-score is particularly useful in imbalanced datasets, as it balances the trade-off between precision and recall.

#### 4.5.7. ROC Curve and AUC

The Receiver Operating Characteristic (ROC) curve plots the true positive rate (sensitivity) against the false positive rate (1—specificity) at various threshold settings. The Area Under the Curve (AUC) provides a single metric to evaluate the model’s performance across all thresholds [44].

- Interpretation: An AUC close to 1 indicates excellent model performance, while an AUC close to 0.5 suggests a model no better than random guessing.

### 4.6. Ethical Considerations

This study utilized publicly available, fully anonymized data obtained from online sources to ensure the protection of sensitive health information. Given the nature of the data, no Institutional Review Board (IRB) approval was required. However, strict ethical standards were upheld by exclusively using datasets that do not contain personally identifiable information, thereby minimizing any risk of re-identification. This approach aligns with best practices for data privacy and security in pediatric health research, ensuring compliance with ethical guidelines for handling sensitive information.

## 5. Results

As we have stated, the data used for this study correspond to the age, gender, and height of children, published by Stanford Medicine Children’s Health. The database contains 1000 records for analysis, results, and conclusions of the study. After a series of pilot tests and adjustments, it was decided to restrict the dataset to children aged 6 to 13 years. However, as we have mentioned, this is a reproducible study, so other datasets can be used in different contexts, such as varying age groups, regions, and countries.

The analysis these data and the construction of the logistic regression model were carried out using the statistical software Rstudio 4.2.3 version [45].

The developed algorithm is freely accessible and will be published in the GitHub repository (https://github.com/).

The result of the logistic regression is as follows:

- Accuracy: 94.65%
- Sensitivity (Recall): 91.03%

These values indicate strong model performance, demonstrating high precision in classification and excellent capability in identifying cases of short stature. The high accuracy reflects the model’s overall effectiveness in correctly predicting both normal and short stature conditions, while the high sensitivity highlights its ability to correctly detect most instances of short stature. This suggests that the model is reliable and effective for applications where identifying growth-related conditions is critical.

**Figure 8:**
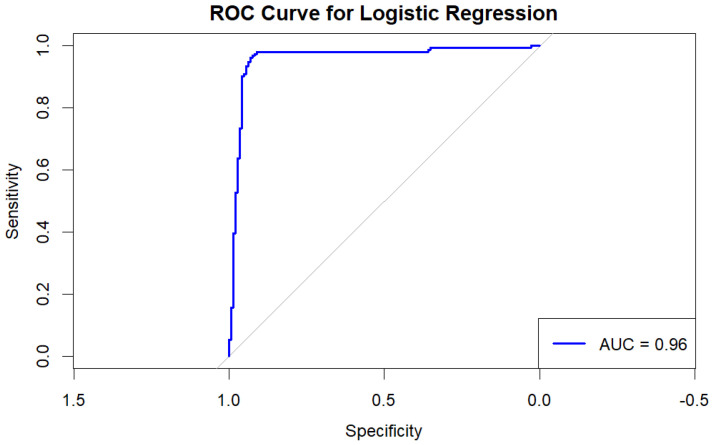
ROC curve for logistic regression.

See Figure 8, which shows the ROC (Receiver Operating Characteristic) curve representing the performance of a logistic regression model with an AUC (Area Under the Curve) of 0.96, indicating excellent predictive capability. This suggests a 96% chance of correctly distinguishing between positive and negative classes, reflecting strong discriminatory power. The curve’s sharp rise towards the top-left corner highlights high sensitivity and specificity, meaning the model effectively identifies true positives while minimizing false positives. The diagonal gray line represents a random classifier (AUC = 0.5), and the curve’s position well above this line confirms the model’s robustness. This outstanding performance makes the model highly reliable for clinical or educational settings where accurate growth assessments are essential. However, despite the impressive AUC, it is important to consider the risk of overfitting, especially when applying the model to new, unseen data.

Despite logistic regression being a powerful and highly interpretable tool for pediatric growth assessment, this study does not delve into feature importance analysis to determine which variables have the greatest influence on the model’s predictions. The inclusion of tools such as LIME (Local Interpretable Model-Agnostic Explanations) and SHAP (SHapley Additive exPlanations) would allow for the decomposition of the contribution of each biometric and demographic variable, providing transparency in clinical decision-making. While LIME offers local interpretations to explain the prediction of a specific case, SHAP assigns global importance values based on game theory, helping to validate the model’s robustness and better understand the most critical risk factors in diagnosing pediatric growth anomalies.

Since the logistic regression model presented in this study achieved an accuracy of 94.65% and an AUC of 0.96, integrating explanatory methodologies such as LIME and SHAP would not only strengthen confidence in the results but also optimize its clinical implementation. These tools would enable healthcare professionals to more intuitively visualize which factors exert the greatest influence on the model’s decisions. By doing so, a balance between predictive accuracy and explainability would be achieved, facilitating the acceptance and adoption of this machine learning-based approach in pediatric medical practice.

### 5.1. Model Interpretability

While logistic regression is a highly interpretable and efficient tool for detecting pediatric growth anomalies, this study incorporates advanced interpretability methodologies such as Local Interpretable Model-Agnostic Explanations (LIME) and SHapley Additive exPlanations (SHAP). These techniques help identify the contribution of each variable to the model’s prediction, enhancing transparency and facilitating its application in clinical settings.

LIME is used to generate local explanations for specific cases, allowing visualization of why the model classified a child within or outside the expected growth range. By analyzing multiple individual observations, it was found that variables such as age, sex, and certain nutritional factors have a substantial influence on classification. This is particularly relevant for clinical practice, as it helps physicians understand the factors leading to a specific decision in a given case.

On the other hand, SHAP was applied to evaluate the global importance of each feature within the model. SHAP values revealed that body mass index (BMI), age, and family history of atypical growth were the most determining factors in predicting abnormal growth. The distribution of SHAP values allowed for visualization of the magnitude and direction of each variable’s impact, helping to establish more precise criteria for future medical evaluations.

### 5.2. SHAP Results

Here are the logistic regression coefficients, which indicate the importance of each variable in predicting pediatric growth anomalies:

- Age: Coefficient of −4.98, suggesting that as age increases, the probability of being classified with an anomalous condition decreases.
- Height: Coefficient of 5.73, indicating that greater height increases the likelihood of a child being classified within the normal growth range. See Table 5.

Machine learning (ML) models have demonstrated superior Interpretability compared to traditional methods in pediatric growth assessment, providing precise and reliable predictions that enhance diagnostic processes [39]. These tools also improve the efficiency of health services by automating data analysis, streamlining workflows, and enabling timely decision-making, ultimately reducing the burden on healthcare professionals [46]. A key strength of ML lies in its ability to detect anomalies in children’s growth patterns, allowing for early interventions and personalized treatment plans that improve long-term outcomes [47]. However, the study emphasizes the importance of addressing contextual variability, as the effectiveness of ML models can differ across school settings, geographical regions, and demographic groups. Developing adaptable and context-sensitive methods is crucial to ensuring widespread applicability and effectiveness. Lastly, future research should focus on overcoming challenges such as data quality, model interpretability, and ensuring robust methodologies. By addressing these issues, ML has the potential to revolutionize pediatric growth assessment, improving health outcomes and service efficiency in diverse clinical and community settings.

The research answers the research question by evaluating how logistic regression algorithms enhance the interpretability of early diagnosis of pediatric growth disorders compared to other machine learning (ML) methods. The hypothesis is verified through the development and analysis of a logistic regression model, whose results show high accuracy (94.65%) and sensitivity (91.03%). Additionally, tools such as LIME and SHAP are used to reinforce the model’s interpretability, allowing healthcare professionals to better understand the factors influencing predictions. These results confirm that logistic regression offers significant advantages in terms of transparency, parsimony, and clinical applicability compared to more complex ML methods.

## 6. Discussion and Conclusions

This study examines the transformative potential of machine learning (ML) in pediatric growth assessment, emphasizing its implications, challenges, and future directions. ML, particularly through logistic regression algorithms, shows significant promise in enhancing diagnostic accuracy and timeliness when identifying growth anomalies [42]. By leveraging clinical and biometric data, these algorithms facilitate early diagnosis and interventions, potentially mitigating the long-term impacts of growth disorders.

The development and implementation of an ML algorithm using the R programming language mark a substantial advancement in this field. Logistic regression is particularly effective for classifying normal versus abnormal growth patterns due to its ability to model binary outcomes [42]. The algorithm achieved an accuracy of 94.65% and a sensitivity of 91.03%, indicating robust performance in detecting short stature conditions. The Area Under the Curve (AUC) of 0.96 from the ROC analysis highlights the model’s strong discriminative ability [44]. However, this raises concerns about potential overfitting, emphasizing the need for external validation with diverse datasets to confirm generalizability.

Despite these promising results, several challenges remain. The success of ML applications heavily depends on the quality and comprehensiveness of input data. Obtaining large, well-annotated, and representative datasets remains a critical hurdle, as pediatric growth assessment requires capturing variability across populations [48]. This often necessitates longitudinal studies. Additionally, stringent data privacy and security measures are essential to ensure compliance with regulations such as GDPR and HIPAA, maintaining public trust and ethical integrity. To address these concerns, this study used publicly available, fully anonymized data obtained from online sources, ensuring the protection of sensitive health information. Given the nature of the data, no Institutional Review Board (IRB) approval was required. Nonetheless, strict ethical standards were upheld by using datasets that do not contain personally identifiable information, minimizing any risk of re-identification. This approach aligns with best practices for data privacy and security in pediatric health research.

Another limitation is that the model’s performance is inherently tied to the geographic and environmental factors of the dataset used for training and validation. Pediatric growth patterns are influenced by variables such as altitude, climate, socioeconomic status, and regional healthcare access, which may limit the model’s generalizability beyond the specific population studied. While the algorithm has demonstrated strong predictive capability, validating its effectiveness across diverse geographic regions is essential to ensure its applicability in broader clinical settings. Future studies should incorporate datasets from different demographic and environmental contexts to enhance the adaptability and robustness of ML-based growth assessment tools.

A significant challenge is the clinical interpretation of ML outputs. Healthcare professionals must understand and trust these models to integrate them effectively into clinical practice [49]. Integrating SHAP into the model analysis allowed for validating the consistency of predictions and enhancing their applicability in clinical settings.

Transparent algorithms and comprehensive clinician training are crucial to bridging this gap [50] and facilitating the adoption of ML-powered growth assessment tools. Moreover, ML models may perform differently across geographic regions and diverse population subsets, necessitating adaptable models that generalize well across various contexts.

While advanced ML models like neural networks and deep learning have gained prominence for their predictive power, logistic regression remains highly valuable due to its simplicity, interpretability, and robustness [3]. Its ability to handle multicollinearity, ease of implementation, and efficiency with small datasets make it particularly advantageous in clinical settings where decision transparency is critical [51].

The findings suggest that while ML significantly improves diagnostic accuracy compared to traditional methods, this enhancement depends on factors such as data quality, model interpretability, and clinical integration. The high performance observed may partially reflect overfitting, reinforcing the need for rigorous external validation to confirm the model’s applicability in diverse clinical environments [42].

In conclusion, ML holds transformative potential in pediatric growth assessment by analyzing complex patterns in large datasets, enhancing diagnostic precision, and improving operational efficiency. However, when applying ML techniques like logistic regression, it is crucial to consider the limitations and assumptions inherent to these models. Logistic regression assumes a linear relationship between independent variables and the log-odds of the outcome, which may not always hold true in pediatric growth data, where relationships can be nonlinear or influenced by interacting factors. Additionally, the model requires independent observations and minimal multicollinearity among predictors. If these assumptions are violated, the model’s performance and interpretability may be compromised. Furthermore, logistic regression is sensitive to imbalanced datasets, which are common in medical studies and may require techniques such as resampling or weighting.

Despite these challenges, when properly validated and applied, logistic regression can serve as a powerful tool within the broader ML framework, uncovering meaningful insights and supporting clinical decision-making in pediatric growth assessment. This study acknowledges key technical challenges in the logistic regression model, including potential overfitting—as indicated by the high accuracy (94.65%) and an AUC of 0.96—necessitating further external validation. Multicollinearity may affect coefficient stability, while the model’s linear decision boundary limits its ability to capture complex growth patterns [52]. Moreover, its binary classification restricts broader diagnostic applications, and its generalizability is constrained by dataset specificity. Addressing these challenges through regularization, external validation, and alternative ML models would enhance robustness and clinical applicability [11].

Fully realizing the potential of ML in pediatric growth assessment requires addressing challenges such as refining dataset quality, ensuring model interpretability, conducting extensive validation studies, and maintaining ethical data management practices. Future research should focus on supporting clinical decision-making and bridging gaps in pediatric care, particularly in regions with limited access to specialized healthcare. Expanding validation efforts across diverse geographic and demographic contexts will also be crucial to ensure model adaptability. By addressing these challenges, ML can play a pivotal role in improving health outcomes for children worldwide [53].

### Implications for Future Research

To fully harness the potential of machine learning (ML) in pediatric growth assessment, it is imperative to address key challenges identified in this study. A primary focus should be enhancing data collection methods to develop large, high-quality, and diverse datasets that represent various populations and growth patterns. Integrating longitudinal data with real-world clinical inputs will significantly improve ML models’ robustness and reliability.

Ensuring data privacy and security is equally critical. Adherence to regulatory frameworks such as GDPR and HIPAA are essential to maintain patient confidentiality and stakeholder trust [54]. Implementing robust encryption, secure data storage, and clear governance policies can mitigate risks associated with data breaches [55].

Developing interpretable and user-friendly ML tools is vital for clinical integration. Models must offer transparent, explainable outputs that healthcare professionals can understand and trust [12]. This includes intuitive interfaces and decision-support systems that facilitate seamless interaction between clinicians and ML algorithms.

Extensive validation studies across diverse populations are necessary to ensure model generalizability and fairness. Cross-institutional collaborations and multi-center trials can identify potential biases and optimize performance across demographic, geographic, and socioeconomic contexts [12].

Future research should explore advanced ML techniques, such as deep learning and ensemble methods, to enhance predictive accuracy [3]. Integrating ML with emerging technologies like wearable health devices and mobile applications may open new avenues for real-time growth monitoring and early intervention, ultimately improving pediatric health outcomes globally.

## Figures and Tables

**Table 1:** Comparative Analysis of Machine Learning Models.

Feature	Logistic Regression	Decision Trees	Random Forest	Support Vector Machines (SVM)	Neural Networks
Model Type	Generalized linear model	Rule-based model	Ensemble of decision trees	Kernel-based classifier	Nonlinear model based on neural layers
Interpretability	High (interpretable coefficients)	Moderate (clear rules, but complexity increases with tree size)	Low (though feature importance can be extracted)	Low (difficult to interpret hyperplanes)	Very low (functions as a “black box”)
Handling of Missing Data	Requires prior imputation	Can handle missing data in some implementations	Can handle missing data in some implementations	Requires prior imputation	Requires prior imputation or specific techniques
Scalability	Good for small to medium datasets	Good for small to medium datasets	Good for medium to large datasets	Scalable but computationally expensive for large datasets	Excellent for large datasets but requires more computational resources
Hardware Requirements	Low	Low to moderate	Moderate	Moderate to high	High (depending on network complexity)
Training Time	Fast	Fast to moderate	Moderate (depends on the number of trees)	Slow (especially with complex kernels)	Slow (especially in deep networks)
Advantages	Simple, interpretable, easy to implement	Easy to interpret, handles nonlinearities	Reduces overfitting, robust, handles nonlinearities	Good generalization, works well with small datasets	High accuracy, captures complex patterns
Disadvantages	Limited to linear relationships, sensitive to outliers	Prone to overfitting in large trees	Less interpretable, higher computational cost	Computationally expensive, difficult to interpret	Difficult to interpret, requires large amounts of data

Source: The authors.

**Table 2:** Height Ranges by Age and Gender (6–13 Years).

Age Group	Min Height—Girls cm	Max Height—Girls cm	Min Height—Boys cm	Max Height—Boys cm
6 years	106.68	124.46	106.68	124.46
7 years	113.03	130.81	113.03	130.81
8 years	119.38	137.16	119.38	137.16
9 years	123.19	143.51	123.825	143.51
10 years	127.00	149.86	128.27	149.86
11 years	133.35	156.21	132.715	155.88
12 years	139.70	162.56	137.16	161.9
13 years	144.78	167.16	143.51	169.37

Source: Stanford Medicine Children’s Health.

**Table 3:** Central Tendency Statistics.

Statistic	Age	Height in Centimeters
mean	9.5	132.4
median	10.0	132.5
std	2.3	14.5
variance	5.1	210.2
min	6.0	88.8
max	13.0	172.9
skewness	−0.03	0.03
kurtosis	−1.22	−0.30

Source: Author’s own creation with information of Stanford Medicine Children’s Health.

**Table 4:** Confusion Matrix.

	Actual Positive	Actual Negative
Predicted Positive	True Positives (TP)	False Positives (PP)
Predicted Negative	False Negatives(FN)	True Negatives (TN)

**Table 5:** SHAP Results.

	Feature	Coefficient
0	Age	−4.984713
1	Height	5.730015

Source: Author’s own creation with study data.

## Data Availability

All data used in the analysis come from public sources. Data for replication are permanently archived is available in the GitHub repository (https://github.com/mrodriguezmarin/ML-pedriatic-growth, accessed on 15 September 2024). All R code necessary to replicate the results of this paper is available in the GitHub repository (https://github.com/mrodriguezmarin/ML-pedriatic-growth). Data may be requested from the corresponding author. Requests will be evaluated on a case-by-case basis and subject to GDPR and other privacy constraints.
